# An Unusual Presentation of Pseudomembranous Colitis

**DOI:** 10.7759/cureus.4570

**Published:** 2019-04-30

**Authors:** Shabana Abdul Jabbar, Sudharsanan Sundaramurthi, TP Elamurugan, Mangala Goneppanavar, Vishnu Prasad Nelamangala Ramakrishnaiah

**Affiliations:** 1 Surgery, Jawaharlal Institute of Postgraduate Medical Education and Research (JIPMER), Puducherry, IND; 2 Pathology, Mahatma Gandhi Medical College and Research Institute, Puducherry, IND

**Keywords:** clostridium difficile, colectomy, embolization, fulminant colitis, rectal bleeding, pseudoaneurysm

## Abstract

Pseudomembranous colitis, also called antibiotic-associated colitis, is caused by the gram-positive anaerobic bacterium *Clostridium difficile* *(C.difficile)*. The infection is common in elderly patients on chronic antibiotic use and in immunosuppressed patients. We report a rare case of pseudomembranous colitis in a 49-year-old male who presented with cramping abdominal pain, abdominal distension, and loose stools, without any pre-existing immunosuppression or chronic drug intake. The computed tomography (CT) picture was suggestive of fulminant ulcerative colitis and the patient underwent total colectomy in view of severe disease. This patient also had rectal bleeding caused by a bleeding pseudoaneurysm of the right internal pudendal artery, which posed diagnostic and therapeutic challenges. Embolization of the pseudoaneurysm was done in the post-operative period. Though the clinical and radiological pictures were suggestive of ulcerative colitis in our patient, this was disproved in the histopathological examination and by the negative serum anti-*Saccharomyces cerevisiae* antibodies (ASCA) testing. The presence of extensive pseudomembranous colitis in this patient masked the bleeding pseudoaneurysm of the internal pudendal artery, as bleeding is a common presentation in fulminant colitis, leading to a delay in the management of the pseudoaneurysm. Such a presentation was not reported in the literature to the best of our knowledge. Considering co-existent pathologies, especially in patients who present with an unobvious clinical picture, can prevent delay in the definitive diagnosis of these conditions.

## Introduction

Pseudomembranous colitis is a serious inflammatory condition of the large bowel characterized by elevated yellow-white plaques that coalesce to form pseudomembranes on the mucosal surface [1]. It is commonly attributed to the bacterium *Clostridium difficile* (*C.difficile*). Long-term use of broad-spectrum antibiotics and a state of immunosuppression are amongst the common risk factors that predispose patients to this condition [2]. The disease commonly affects the middle-aged and the elderly. Overall, this condition is fairly common in India with a reported incidence of about 15%-20% among patients taking antibiotics [3]. Patients with pseudomembranous colitis usually present with a mild cramping abdominal pain, fever, and diarrhea and few acute cases progress to fulminant colitis (2%-8%) leading to toxic megacolon, perforation, and bleeding [4]. The latter has a more dramatic presentation of severe abdominal pain, rectal bleeding, and shock. Here we present such a case of pseudomembranous colitis with rectal bleeding complicated by a bleeding pseudoaneurysm mimicking fulminant inflammatory bowel disease. There were further points of interest in that the patient had no history of hospitalization, immunosuppression or antibiotics use in the recent past, hence, delaying the diagnosis.

## Case presentation

A 49-year-old male patient was admitted with complaints of cramping left lower abdominal pain, abdominal distension, and loose stools with the passage of blood and pus per rectum, associated with high-grade fever for 10 days. He had no previous history of altered bowel habits or chronic drug intake. On examination, he had an initial pulse rate of 110/min and blood pressure of 90/60 mm Hg. He was febrile and clinically pale at presentation; the abdominal examination revealed tenderness and guarding in the bilateral iliac fossae. On digital rectal examination, the rectum was filled with blood clots, the mucosa was friable, and a doubtful defect obscured by clots in the posterior wall of the rectum was felt, suggesting a perforation.

**Figure 1:**
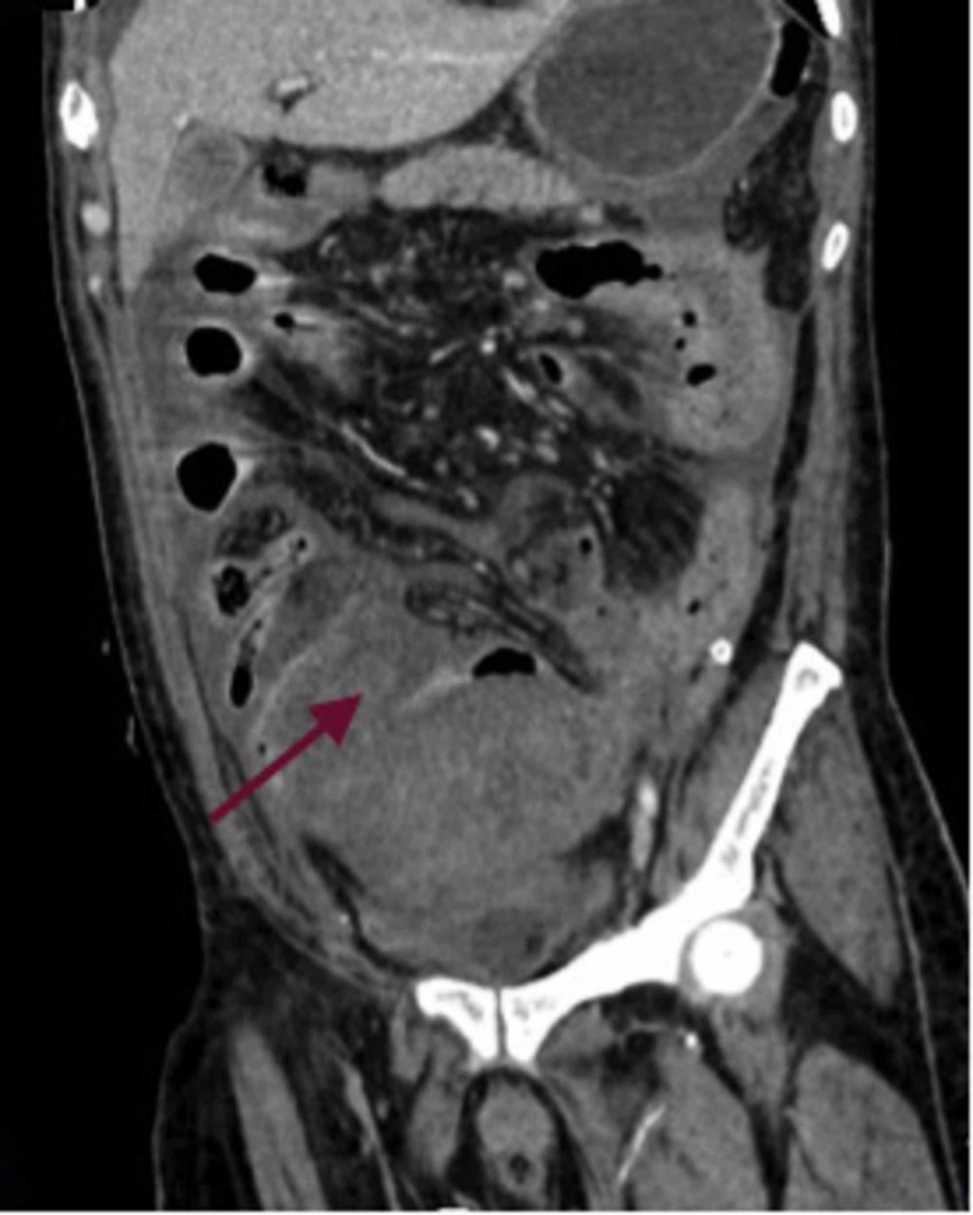
Coronal view contrast-enhanced computed tomography (CECT) of the abdomen showing pancolonic oedema (arrow)

Baseline blood investigations showed anemia with a hemoglobin of 7 g/dl and elevated white blood cell count of 20,000/mm^3^. He was stabilized with intravenous fluids, blood products, and was started on broad-spectrum antibiotics. A contrast-enhanced computed tomography (CECT) abdomen was done, which showed diffuse pan-colonic edema (Figure 1).

A 0.5 x 0.5 cm perforation was visualized at the rectosigmoid junction. A hyperdense collection was noted posterior to the perforation and anterior to the pre-sacral fascia, suggestive of a hematoma. A pseudoaneurysm was noted in a branch of the right internal iliac artery but there was no active blush.

**Figure 2:**
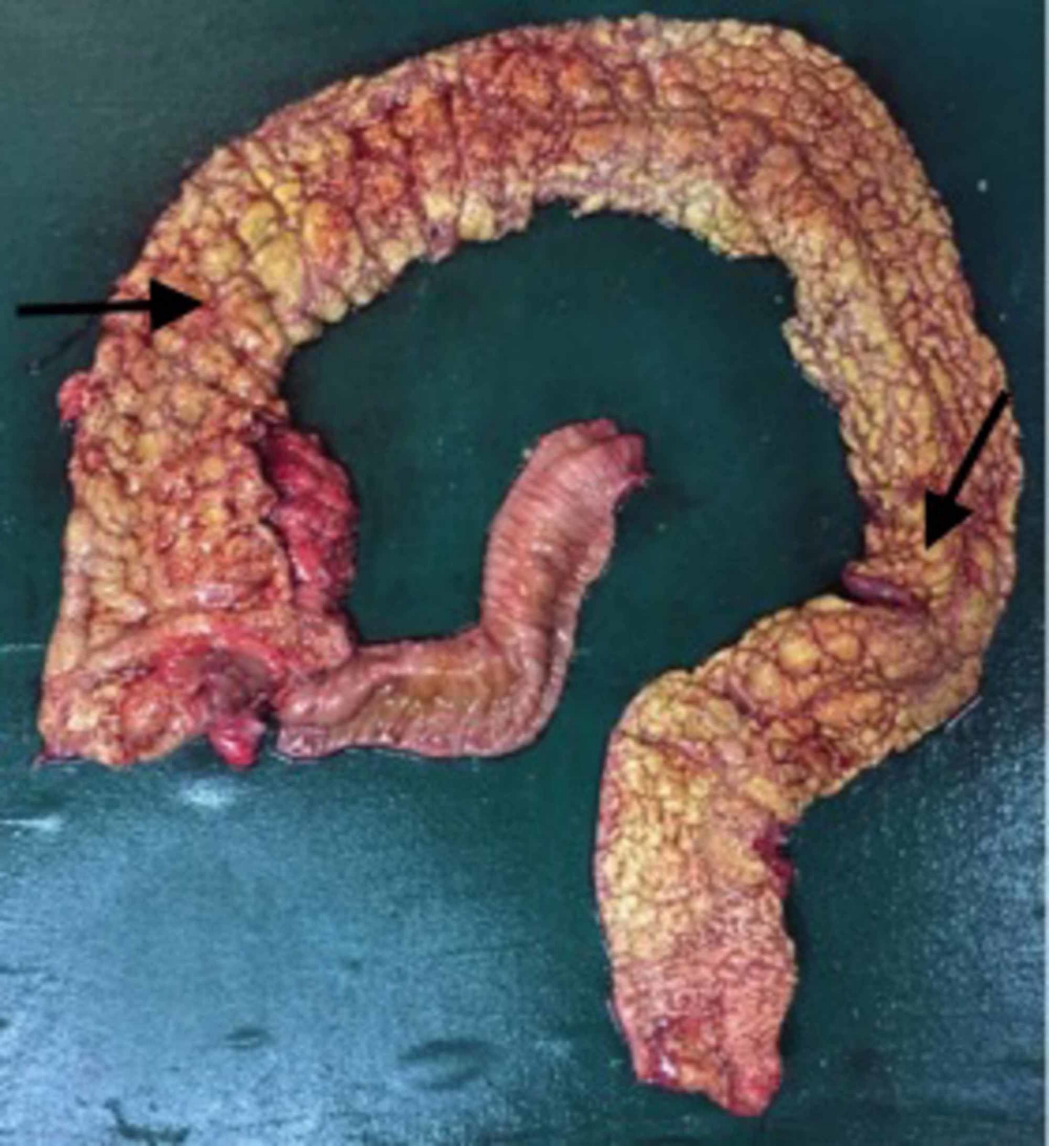
Proctocolectomy specimen showing diffuse mucosal exudates and pseudo polyps (marked by arrows)

With these findings suggesting a diagnosis of fulminant ulcerative colitis with perforation, and in view of the ongoing blood loss, unstable vitals, high-grade fever, the patient was taken up for emergency laparotomy. Intra-operatively, there was pus collection in the pelvis with an upper rectal perforation and the entire colon was edematous and congested. Subtotal proctocolectomy with Hartman’s procedure and end ileostomy were done. The colonic mucosa was found to be studded with yellowish granular and thickened areas (Figure 2).

The rectal stump with a posterior wall perforation was sutured and the area was drained. In view of the pseudoaneurysm finding on CECT, a thorough search was done for any source of bleeding and no active bleeding was noted.

The patient was given multiple blood transfusions and he showed initial improvement in the early post-operative period. However, he developed high spiking fever and persistent pus and blood-stained discharge per rectum from postoperative day three. He was started on piperacillin-tazobactam based on the culture sensitivity report of the pus, which showed growth of *Escherichia coli*. The bleeding per rectum was attributed to lysis of the remaining hematoma.

However, the patient continued to have febrile spikes in spite of higher antibiotics. On postoperative day five, re-exploration of the abdomen was done to look for any remaining infective foci or pus collection. Intra-operatively a pus pocket was found tracking along the previous drain site to the parietal wall. It was drained and thorough lavage was given. Three drains were placed, one each, within the rectal stump, the parietal wall pus pocket, and in the abdominal cavity. There was no active bleeding source noted in the second laparotomy.

**Figure 3:**
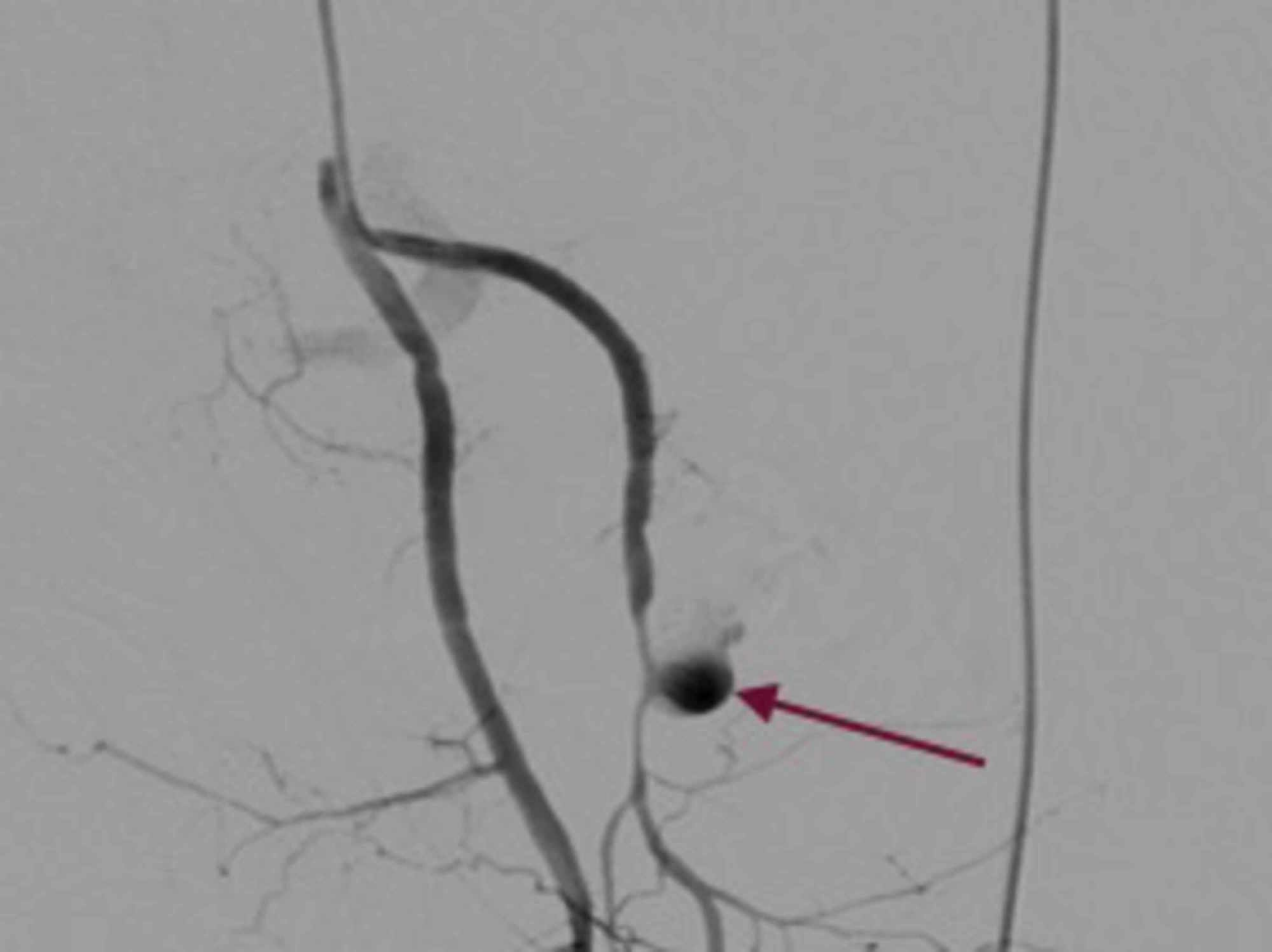
Computed tomography (CT) angiography showing right internal pudendal artery aneurysm (arrow)

On postoperative day 11, the patient had profuse bleeding per rectally and the rectal stump drain showed fresh blood. A computed tomography (CT) angiogram was done, which revealed a pseudoaneurysm of size 3 x 1.2 cm, originating from the right internal pudendal branch of right internal iliac artery adjacent to the pyriformis muscle on the right side (Figure 3).

**Figure 4:**
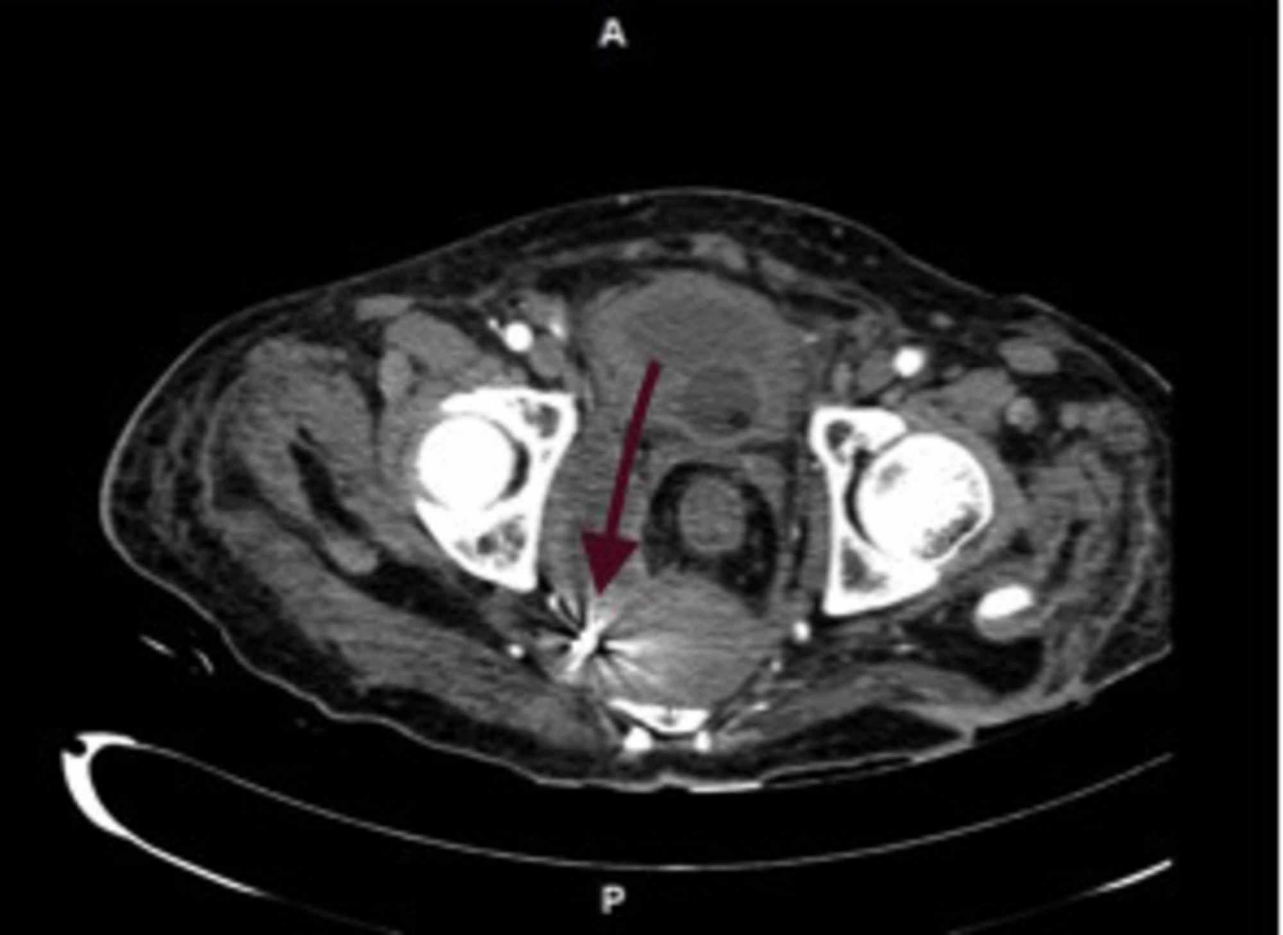
Post-embolization of the right internal pudendal artery aneurysm (embolized coil pointed by the arrow)

There was diffuse iso to hyperdense foci surrounding the aneurysm, suggesting a haemorrhagic clot. The aneurysm was embolised under vision (Figure 4).

Post-procedure, his rectal bleed reduced and gradually became nil over the next four days. His blood parameters improved and hemoglobin continued to remain stable.

**Figure 5:**
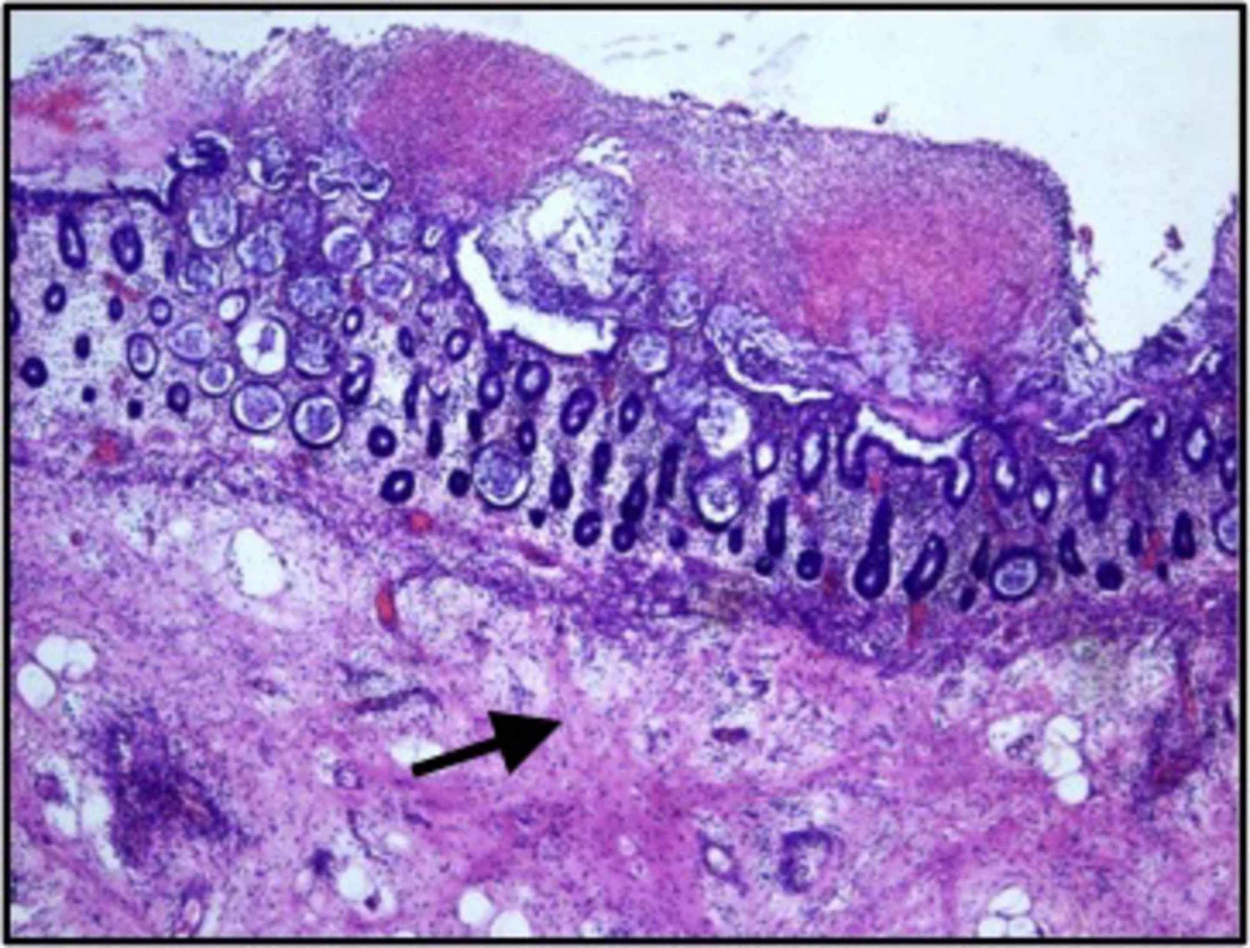
Histopathological examination showing mucosal exudates with unremarkable sub mucosa (arrow pointing to normal submucosa) (Hematoxylin & eosin; magnification 40x)

**Figure 6:**
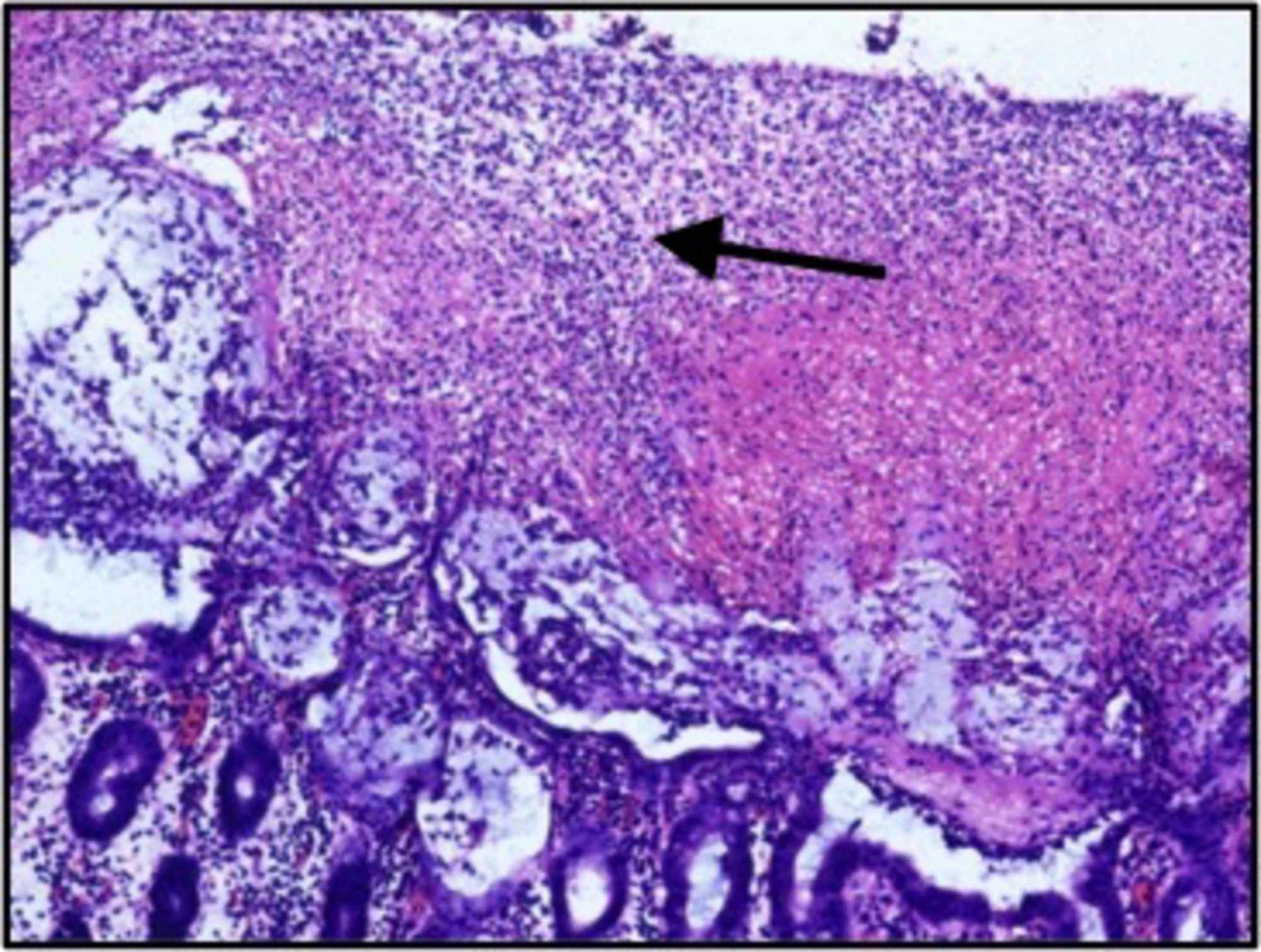
High power histological view showing mucosal ulceration which is covered with sheets of neutrophils (arrow showing sheets of neutrophils) (Hematoxylin & eosin; magnification 100x)

Post-operative histopathological examination of the colectomy specimen showed a picture of pseudomembranous colitis. Gross findings of mucosal exudates with unremarkable submucosa and microscopically, the presence of mucosal ulceration covered with sheets of neutrophils were seen (Figures 5-6).

The patient was discharged on postoperative day 16 following the second surgery. The patient underwent Hartman reversal with ileorectal anastomosis after three months.

## Discussion

Pseudomembranous colitis is most commonly caused by *C.difficile* infection. It is rarely, also caused by other bacteria like *Escherichia coli* and *Klebsiella spp*. Viral colitis by cytomegalovirus, ischemic colitis, collagenous colitis, inflammatory bowel disease, and various drugs are also implicated in the etiopathogenesis [5-6]. Usually, patients presenting with this condition have a pre-existing history of chronic drug intake, altered bowel habits or long-term immunosuppression. However, there has also been few reported cases of pseudomembranous colitis, with none of the above-mentioned factors and wherein, the cause was postulated to be an infection that complicated colonic obstruction or long-standing debilitating disease [7].

Patients with this disease commonly present with a mild to moderate disease. Their symptoms range from diarrhea, abdominal pain and fever to rarely abdominal distension and rectal bleeding [8]. The latter symptoms are attributed to a more fulminant presentation of pseudomembranous colitis which, if untreated or unresponsive to medical management can lead to a complicated disease with toxic megacolon or colonic perforation. This is a less common presentation of this disease, with the incidence of around 2%-8%. These cases of the complicated disease require colectomy and have high mortality of 38%-80 %, which is more in the elderly age group and hence early surgical intervention is recommended in these patients [9].

Pseudomembranous colitis caused by Cl. difficile infection is diagnosed by stool testing. The most commonly used tests are *C. difficile* cytotoxin neutralization assay (CCNA) and toxin A+B enzyme immune assays [10]. Nucleic acid amplification tests with polymerase chain reaction (PCR) on rectal swab specimens and glutamate dehydrogenase (GDH) detection in stools are newer advances in the diagnosis of *C.difficile* infection. However, these tests are recommended in patients who present with an uncomplicated disease [11]. The patient in our scenario, had no history predisposing him to *C.difficile* infection. While his CT imaging showed features of fulminant ulcerative colitis, a possibility of carcinoma rectum was also considered, as there were large firm blood clots on per rectal examination, indicating the probability of an ulcero-proliferative lesion in the rectum or colon. However, the patient was promptly taken up for surgery without causing a delay in the management of his condition.

Cases of pseudomembranous colitis complicating ulcerative colitis have been reported in the past. Wang et al. reported a case of pseudomembranous colitis in a patient with long term ulcerative colitis, after receiving clindamycin for infectious colitis [12]. A similar case of antibiotic-associated pseudomembranous colitis was also reported by Kawaratani et al. in a woman who had received antibiotics for a post-operative infection and was already on steroid treatment for ulcerative colitis [13]. History of immunosuppression and use of antibiotics is common to both these cases, unlike in our patient. The diagnosis of ulcerative colitis in the patient in our report was a clinical and radiological one and it was later disproved after the histopathology report and a negative serum anti-*Saccharomyces cerevisiae* antibodies (ASCA) report.

The confirmatory diagnosis of pseudomembranous colitis is made by visualization of pseudomembranes on the colonic mucosa. The post colectomy specimen in this patient showed diffuse mucosal exudates and normal serosal surface with the absence of creeping fat. On histopathological examination, mucosal exudates with unremarkable submucosa and mucosal ulceration covered with sheets of neutrophils were seen.

The patient also had persistent rectal bleeding which was attributed to the primary pathology in the remnant rectal stump. There was no suspicion of a bleed from the pseudoaneurysm as the CT imaging had shown no active blush and also, there were no intra-operative signs suggestive of the same in the first two surgeries. The bleed was due to rupture of a branch of the internal iliac artery forming a hematoma, which led to rectal bleed through the posterior wall perforation in the rectal stump. This is a rare occurrence and no similar case of pseudomembranous colitis complicated by aneurysmal bleed has been reported earlier. The presence of this co-existing abnormality posed a diagnostic dilemma in this patient and also led to a delay in the management of the bleeding. This case report hence stresses the importance of a thorough study of radiological images and need for repeat imaging in any patient with clinically unexplainable parameters, in order not to miss out other coexisting pathologies. Any uncontrolled or persistent bleeding per rectum should at once prompt a search for other sources of bleed even in the presence of more obvious pathology. This will prevent loss of valuable time and optimize management and reduce the duration of hospital stay.

While considering differentials for complicated cases of colitis, the possibility of pseudomembranous colitis should be kept in mind along with inflammatory bowel disease. Thus, a high index of suspicion is necessary. This will direct investigations towards a definitive diagnosis. For consideration, a stool antigen test for *C.difficile* would have resulted in earlier diagnosis of pseudomembranous colitis in this case. This would have helped start appropriate therapy earlier in the course of the disease. As opposed to this, the definitive diagnosis arrived only at a later stage in the post-operative period after the histopathology report was available. This delay can be avoided by considering concomitant pathologies, as in this case, the possibility of ulcerative colitis complicated by superimposed *C.difficile* infection.

The importance of considering the possibility of multiple coexisting lesions that create an unobvious picture cannot be more emphasized.

## Conclusions

Acute fulminant colitis is a rare presentation in pseudomembranous colitis and can mimic inflammatory bowel disease. The diagnosis is challenging in patients who do not have previous lower gastrointestinal complaints, especially when they do not have factors predisposing them to pseudomembranous colitis. In cases of persistent bleeding, other sources of bleed should be considered even in the presence of a more obvious pathology to prevent loss of valuable time and to optimize management.
